# Dispersion of Heat Flux Sensors Manufactured in Silicon Technology

**DOI:** 10.3390/s16060853

**Published:** 2016-06-09

**Authors:** Katir Ziouche, Pascale Lejeune, Zahia Bougrioua, Didier Leclercq

**Affiliations:** Institute of Electronics, Microelectronics and Nanotechnology, University Lille 1 and CNRS; Villeneuve d’Ascq, 59652, France; pascale.lejeune@univ-lille1.fr (P.L.); zahia.bougrioua@iemn.univ-lille1.fr (Z.B.); didier.leclercq@univ-lille1.fr (D.L.)

**Keywords:** silicon, sensor, CMOS, heat flux, thermoelectric, dispersion

## Abstract

In this paper, we focus on the dispersion performances related to the manufacturing process of heat flux sensors realized in CMOS (Complementary metal oxide semi-conductor) compatible 3-in technology. In particular, we have studied the performance dispersion of our sensors and linked these to the physical characteristics of dispersion of the materials used. This information is mandatory to ensure low-cost manufacturing and especially to reduce production rejects during the fabrication process. The results obtained show that the measured sensitivity of the sensors is in the range 3.15 to 6.56 μV/(W/m^2^), associated with measured resistances ranging from 485 to 675 kΩ. The dispersions correspond to a Gaussian-type distribution with more than 90% determined around average sensitivity $\bar{S_{e}}$ = 4.5 µV/(W/m^2^) and electrical resistance $\bar{R}$ = 573.5 kΩ within the interval between the average and, more or less, twice the relative standard deviation.

## 1. Introduction

Heat flux sensors allow to obtain a direct reading of the thermal transfers between a surface and its environment in a real-time manner. The balance of exchanged heat (received or supplied) that can be conductive, convective and radiative is expressed by means of the measured thermal flux (in W·m^−2^). The design requirements of the flux sensor are a very low thickness associated with a good thermal conductivity to be representative of the exchange between the surface on which the microsensor is placed and its surrounding environment. To fulfill these requirements and to envisage a large-scale development, we developed sensors in CMOS-compatible technology on silicon wafers which thickness is typically lower than 400 μm and the thermal conductivity is pretty high (*λ_th_* = 140 W/m·K). Another advantage of the microsensors we present lies in the simple relation between the thermal flux and the corresponding DC voltage measured by the thermopile of the heat flux sensor [1,2,3,4]. These sensors can be used in a large range of applications: contactless temperature measurement [4,5,6,7], evaporation of latent heat [8], and determination of dissipated thermal power [9]. Information related to the manufacturing process of the system is detailed in [10].

In this paper, we targeted a fine study of the sensor’s reliability. The two main parameters that qualify the fabrication reliability of these large-area sensors (typically 5 × 5 mm^2^) on a 3-in full plate are the sensitivity and the electrical resistance of the heat flux microsensor. The optimization of the fabrication process requires a fine control of the thicknesses, electric resistivities and thermoelectric powers of the thermoelectric materials. They are measured at each technological fabrication step. In spite of the limits of the 3-in technology, a very good reliability of our manufacturing process has been achieved with an associated Gaussian dispersion of the sensitivity and electrical resistance values. We also show that the measured sensitivities are in a good agreement with those computed by a dedicated mathematical model [10]. In Section 2, we detail the design of the sensor and the modelling of the structure. The fabrication of the sensor, related experiments and the discussion are then presented in the Section 3.

## 2. Sensor Design and Modeling

### 2.1. Sensor Description

**Figure 1:**
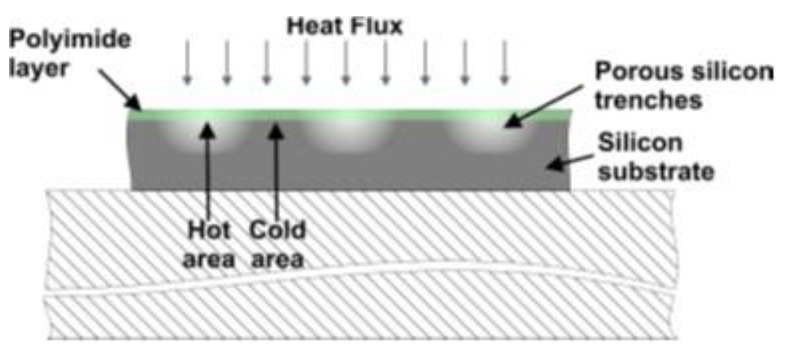
Cross-sectional view of periodic temperature variations induced by the heat flux.

The originality of the sensor consists of a thermal asymmetry due to the use of porous silicon trenches in a silicon wafer. Compared to silicon (*λ_Si_* ~140 W/m·K), porous silicon presents a thermal conductivity 100 times lower (*λ_Porous__Si_* ~1.2 to 2 W/m·K) [11,12,13]. When a transverse heat flux *ϕ* flows through the sensor, this asymmetry leads to lateral periodic temperature variations ΔT inside the sensor (Figure 1).

**Figure 2:**
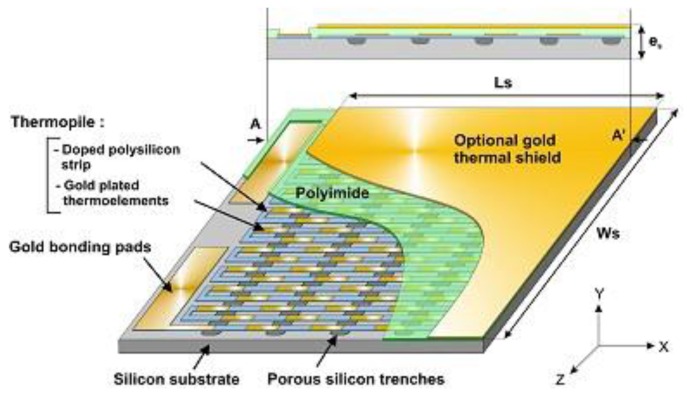
Schematic diagram of the heat flux microsensor.

A gold/polysilicon thermopile, made of N thermocouples correctly arranged as shown in Figure 2, transforms these gradients of temperature into a Seebeck voltage:

(1) V=NαΔT

where *α* (µV/K) is the Seebeck coefficient of the thermocouples.

Assuming the Fourier law [14], a coefficient *r_th_* considered as a two-dimensional (2D) thermal resistance between two successive junctions can be expressed by

(2) $$r_{\text{th}}=\frac{\Delta T}{\varphi }(Km^{2}/W)$$

This thermal coefficient is a function of the structural dimensions and thermal conductivities of the different parts and layers constituting the sensor and is determined by using numerical modelling [10]. The sensitivity of the microsensor to a heat flux is given by

(3) $$S_{e}=\frac{\text{dV}}{d\varphi }={N\alpha r}_{\text{th}}=\alpha \frac{A_{S}}{\left(w+i\right)}\frac{r_{\text{th}}}{L}(Vm^{2}W^{-1})$$

where *A_s_* is the surface of the sensor, *L* is the length of a thermocouple, *w* and *i* are, respectively, the width of the strips and interstrip of the thermopile (Figure 2).

The electrical resistance *R_el_* of the thermopile which is made of a polysilicon track partially covered by gold strips is

(4) $$R_{\text{el}}=N\left(\frac{\rho_{\text{poly}}L}{2e_{\text{poly}}w}+\frac{\rho_{\text{poly}}\rho_{\text{Au}}L}{2(\rho_{\text{Au}}e_{\text{poly}}+\rho_{\text{poly}}e_{\text{Au}})w}\right)\;\;\;(\Omega )$$

with *e_poly_*, *e_Au_* and *ρ_poly_*, *ρ_Au_*, respectively, the thicknesses and the electrical resistivities for the polysilicon tracks and gold strips.

### 2.2. Thermal Modeling

A numerical model was developed, using COMSOL multiphysics software (COMSOL™ Multiphysics), to optimize the geometrical dimensions of the sensors [10].

**Figure 3:**
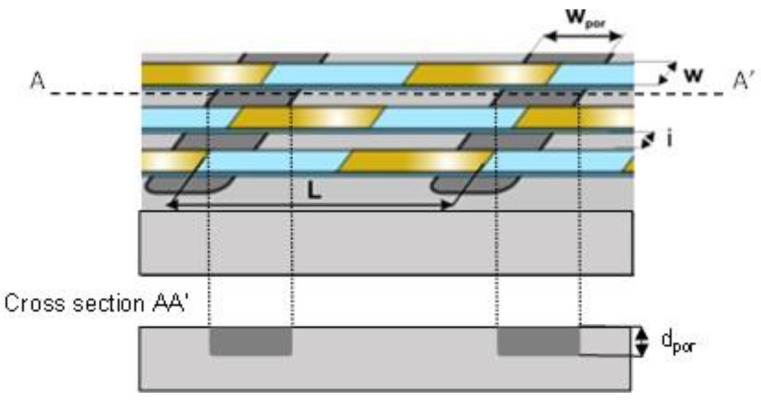
Zoom on an elementary cell of the heat flux microsensor.

As shown in Equation (3), the sensitivity to the heat flux density, *S_e_*, is proportional to *r_th_*/*L.* So, to determine the optimal width of the porous trenches *w_por_* according to the thermocouple length *L* (Figure 3), the evolution of *r_th_*/*L* is studied *versus* the ratio *w_por_*/*L* (from 0 to 1) for different values of lengths *L* (from 100 to 1000 µm) and depths *d_por_* (from 50 to 300 µm). It is demonstrated that the maximum values of *r_th_*/*L* are systematically obtained for the same ratio *w_por_*/*L* = 0.9, whatever the depth of the porous silicon box and the length of the thermocouple.

**Figure 4:**
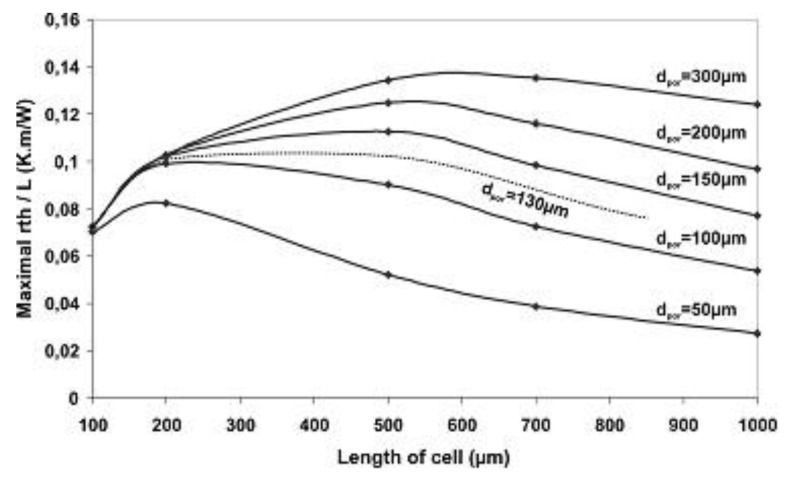
Values of *r_th_*/*L versus* cell length for different depths of porous silicon trenches for *w_por_*/*L* = 0.9.

In these conditions, the maximum values of *r_th_*/*L* as a function of the cell length *L* for different depths *d_por_* of porous silicon boxes are represented in Figure 4.

One can observe that the maximum value of *r_th_*/*L* (0.14 K·m/W) that corresponds to an optimal sensitivity, is obtained for *L* = 500 µm and *d_por_* = 300 µm. In fact, in practice, the depth *d_por_* cannot exceed 150 µm because of the weak mechanical resistance of porous silicon. For a sensor with a width set to 5 mm, the optimum values of the widths of the strips and interstrips of the thermopile are, respectively, *w* = 80 µm and *i* = 20 µm. The corresponding polysilicon thickness is *e_poly_* = 0.6 μm.

So, to summarize, the geometrical dimensions kept to fabricate the microsensors on a 3-in wafer are: *L* = 500 µm, *e_poly_* = 600 nm, *w* = 80 µm and *i* = 20 µm.

## 3. Experimental Results and Discussion

### 3.1. Sensor Fabrication

**Figure 5:**
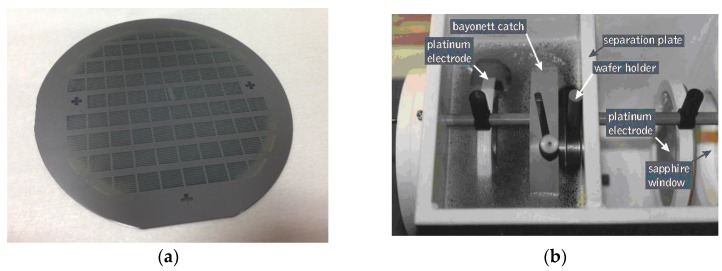
(**a**) Photography of a 3-in-diameter silicon wafer with processed porous silicon trenches; (**b**) Double cell HF tank for porous silicon etching with electrolytic backside contact (AMMT^™^).

The sensitivity *S_e_* depends mainly on *d_por_* and *w_por_* (the depth and width of the porous silicon boxes) and on α (the Seebeck coefficient of thermocouples). The electrical resistance *R_el_* depends primarily on the thicknesses *e_poly_*, *e_Au_* and electrical resistivities *ρ_poly_*, *ρ_Au_* of the polysilicon track and gold strips of the thermoelectric layer (Equation (4)). Thus, these parameters were particularly controlled during the fabrication process. The porous silicon trenches are processed onto 3-in-diameter <100> silicon wafers (thickness is 380 ± 25 µm, p-type doping with Bore and electrical resistivity between 0.009 and 0.01 Ω.cm). The wafers were patterned and anodized (Figure 5a) in a double cell tank (Figure 5b) during several minutes in a mixture of 27% fluorhydric acid (HF), 38% water and 35% ethanol with a current density of 100 mA/cm^2^ [15].

The engraving speed is approximately 4 to 5 µm per minute. The anodization of silicon lasts about 25 min, resulting in boxes with depths that varies from 100 µm on the center of the wafer to 130 µm on the edges (measured by scanning electron microscopy). These edge effects are mainly due to the dimensions of the wafer (3 in) which are smaller than those of the electrodes (4 inch, Figure 5b). So, the electric lines of current which pass from an electrode to the other one through the wafer undergo a deviation which generates a stronger concentration of the lines in the periphery of the wafer, locally inducing the over-engraving. The polysilicon layer was *in situ* n-type doped with Phosphorus during its deposition by LPCVD (low pressure chemical vapor deposition). The thermopile zigzag-shaped track was realized by lithography and mesa etching using reactive ion etching with SF_6_ and CF_4_ mixture gas. The periodical plated thermoelements were processed by lift-off techniques using the evaporation of a Ti/Au bilayer. The in-plane electrical properties of the polysilicon layer were characterized by Van der Pauw and Hall effect methods. The Seebeck coefficient of the thermocouple was measured on equivalent layers with an experimental set-up [16].

Table 1 presents the range of values for the electrical resistivity *ρ_poly_*, thermoelectric coefficient *α* and thickness *e_poly_* of the polysilicon layer measured in different locations of the wafer [16]. The thickness *e_Au_* of the Ti/Au bilayer is 250 nm ± 10 nm. Because of its very low electrical resistivity, this layer has a minor impact on the value of the electrical resistance of the thermopile (Equation (4)).

### 3.2. Sensor Characterizations

A set of five masks was used for the fabrication of the sensors on a complete 3-in wafer. First of all, the positions of the efficient heat flux sensors on the wafer were established. Then, the sensitivities and electrical resistances of the 74 efficient sensors were measured: the values are given in Table 2. The sensitivities *S_e_* of the heat flux sensors were determined by the radiative method. The calibration is described in [10]. These sensors do not need cooling. They can operate from ambient temperature up to 200 °C.

A first analysis of these results shows that, for the four sensors situated at the four corners of Table 2 (nbr 1, nbr 6, nbr 69 and nbr 74), the sensitivity *S_e_* is very low because of incomplete porous silicon boxes, as shown in Figure 5a. This is caused by the mark left by the seal glued onto the wafer during the electrolysis. The seal is used to isolate the electrolytes of the two tanks in order to avoid electric current leaks. One can also find a few other low values of electrical resistances that are due to contacts resulting in shunts between two adjacent strips of the thermopile (nbr 44, nbr 52 and nbr 65). These kinds of problems can occur during different steps in the fabrication process: lithography, polysilicon engraving or lift-off of the Ti/Au layer which is the second material of the thermopile. We can mention here the challenge of fabricating 500 strips that are 5 mm long and spaced 20 µm apart.

**Figure 6:**
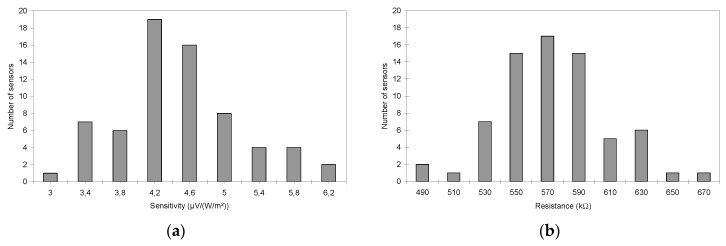
Dispersion histograms of (**a**) sensitivities; and (**b**) resistances.

When only considering the 67 reliable sensors, one finds that the measured sensitivities vary between 3.15 and 6.56 µV/(W/m^2^) and, that the measured resistance values lie between 485 and 675 kΩ. The average values of sensitivity and electrical resistance are respectively $\bar{S_{e}}\;$= 4.5 µV/(W/m^2^) and $\bar{R}$ = 573.5 kΩ. The global dispersions of both parameters are given in Figure 6. The two histograms exhibit Gaussian-type distributions. From these results the calculated standard deviations are, respectively, *σ_Se_* = 0.74 µV/(W/m^2^) and *σ_R_* = 34.8 kΩ. Furthermore, 95.6% of the sensitivity values are within the interval [$\bar{S_{e}}\;$*− 2σ_Se_;* $\bar{S_{e}}\;$ *+ 2 σ_Se_*] and 73.5% in *[*$\bar{S_{e}}\;$ *− σ_Se_;* $\bar{S_{e}}\;$ *+ σ_Se_]*. Similarly, 94.3% of the resistances are in the range [$\bar{R}$*− 2σ_R_;* $\bar{R}$ *+ 2σ_R_*] and 74.3% in [$\bar{R}$ *− σ_R_;* $\bar{R}$*+ σ_R_*].

### 3.3. Discussion

As stated before the depth of the porous silicon boxes *d_por_* is between 100 µm and 130 µm. The corresponding thermal coefficient *r_th_* is deduced from numerical model curves (Figure 4) and the ratio *r_th_*/*L* lies between 0.088 and 0.101 K·m/W.

By introducing these values of *r_th_*/*L* and the thermoelectric coefficient α given in Table 1 in Equation (3), the calculated sensitivity can be evaluated between 4.84 and 6.57 µV/(W/m^2^). So the calculated sensitivity range cross the experimental range *[*$\bar{\;S_{e}}\;$ *− σ_Se_;* $\bar{S_{e}}\;$ *+ σ_Se_]* (*i.e.*, 3.76–5.24 µV/(W/m^2^)) and encompass the experimental average value (4.5 µV/(W/m^2^)). There is a slight shift in the measured values.

In the same way, the electrical resistances are calculated by using Equation (4). The contact resistances between the polysilicon layer and the (Ti/Au) bilayer are measured by the transmission line method (TLM) and the Van Der Pauw method. It is approximately 7 Ω for each thermocouple, corresponding to few kΩ for the sensor, and it can therefore be neglected. Consequently, with the minimal and maximal values of *ρ_poly_* and *e_poly_* given in Table 1, the theoretical values of the total resistance vary between 517 and 658 kΩ. The comparison to the measured values, which range between 538.7 and 608.3 kΩ (average value of 573.5 kΩ), is quite good.

The good agreement observed between the theoretical and measured values demonstrates the performance and the reliability of the fabrication process. Actually the electrical resistances and sensitivities of the sensors are higher at the periphery of the wafer. The values of the sensitivities are explained by the greater thickness of the porous silicon boxes and the higher thermoelectric power at this location of the wafer. A lower sensitivity for some peripheral sensors is due to an incomplete manufacturing of the corresponding porous silicon boxes. Concerning the values of the electrical resistances, the variations are essentially due to a local lower thickness of the polysilicon and to a higher resistivity.

## 4. Conclusions

In this paper, a study of the performance and reliability of heat flux microsensors fabricated by means of technological processes and equipment suited to 3-in wafers has been proposed. In particular, we show that the dispersion observed in terms of sensitivity and electrical resistance is closely related to the limits of the equipment and of the processes used. Actually, the 3-in processes do not allow obtaining homogeneous coats on 3-in wafers (two sizes above needed). The LPCVD technique allows achieving a thickness of polysilicon with fluctuations of about ±5%. The resistivity and the thermoepower bound to the doping level are not homogeneous, with fluctuations of about ±8%. A doping by implanting should allow a better homogeneity than the doping *in situ*. The anodization process entails a difference in the thickness of porous silicon between the center and the periphery of the wafer which is translated to a difference in sensitivity of 10%. This value can be reduced by increasing the distance separating the electrodes of the wafer.Finally, it has been shown that differences between the values of sensors’ sensitivities in the periphery of the wafer are mainly due to edge effects. If we consider the results obtained for the sensors located in an area of about 2-in in diameter centred on the wafer, the dispersion is much better: with 80% of the sensitivities within the interval $\bar{S_{e}}$-*2σ_Se_;*$\bar{S_{e}}$* + 2σ_Se_* where *Se* = 4.46 µW/(W/m^2^) and *σ_Se_* = 0.44 µW/(W/m^2^). These results altogether highlight the reliability and maturity of the technology process. The heat flux microsensors are therefore a viable solution for applications outside a laboratory environment.

## Figures and Tables

**Table 1:** Range of values for electrical resistivity *ρ_poly_*, thermoelectric coefficient *α* and thickness *e_poly_* of the polysilicon layer.

	*ρ_poly_* (mΩ·cm)	α (µV/K) at 298 K	*e_poly_* (nm)
Dispersion	0.0205–0.024	220–260	570–620

**Table 2:** Sensors’ sensitivities and electrical resistances measured on a complete 3 in wafer.

	Column	*1*	*2*	*3*	*4*	*5*	*6*	*7*	*8*	*9*	*10*
Ligne											
***1***	*Sensor number*			***1***	***2***	***3***	***4***	***5***	***6***		
	*R (kΩ)*			637	570	580	675	570	557		
	*Se (µV/(W/m²))*			2	3.9	3.39	3.9	3.23	2.02		
***2***	*Sensor number*		***7***	***8***	***9***	***10***	***11***	***12***	***13***	14	
	*R (kΩ)*		548	560	570	570	589	657	620	584	
	*Se (µV/(W/m²))*		4.59	3.47	5.44	4.5	4.2	4.15	3.44	3.32	
***3***	*Sensor number*	***15***	***16***	***17***	***18***	***19***	***20***	***21***	***22***	***23***	***24***
	*R (kΩ)*	577	573	571	569	590	550	530	588	625	597
	*Se (µV/(W/m²))*	3.54	5.73	3.97	3.91	3.86	4	4.5	4.63	4.76	3.24
***4***	*Sensor number*	***25***	***26***	***27***	***28***	***29***	***30***	***31***	***32***	***33***	***-***
	*R (kΩ)*	547	570	550	538	620	570	565	612	590	
	*Se (µV/(W/m²))*	6.46	5.3	4.78	4.12	3.9	4.17	4.12	4.3	4.46	
***5***	*Sensor number*		***34***	***35***	***36***	***37***	***38***	***39***	***40***	***41***	
	*R (kΩ)*		552	435	530	545	490	573	580	620	
	*Se (µV/(W/m²))*		6.13	4.3	4.45	4.1	4.12	4.29	4.64	5.24	
***6***	*Sensor number*	***42***	***43***	***44***	***45***	***46***	***47***	***48***	***49***	***50***	***51***
	*R (kΩ)*	537	535	278	554	535	525	485	596	604	620
	*Se (µV/(W/m²))*	6.56	5.57	2.35	4.54	4.19	4.23	4.2	4.23	4.49	6.02
***7***	*Sensor number*	***52***	***53***	***54***	***55***	***56***	***57***	***58***	***59***	***60***	***61***
	*R (kΩ)*	136	556	556	564	531	546	555	606	588	585
	*Se (µV/(W/m²))*	3	5.57	4.56	4.38	4.1	4.15	4.53	4.35	4.94	5
***8***	*Sensor number*		***62***	***63***	***64***	***65***	***66***	***67***	***68***	***-***	
	*R (kΩ)*		550	575	561	350	585	585	605		
	*Se (µV/(W/m²))*		5.84	4.49	4.49	2.8	4.9	4.9	4.24		
***9***	*Sensor number*			***69***	***70***	***71***	***72***	***73***	***74***		
	*R (kΩ)*			557	516	574	584	598	600		
	*Se (µV/(W/m²))*			2.35	5.7	5.2	5.2	4.52	1.57		
